# The role of musical pitch in long‐distance defensive signaling

**DOI:** 10.1111/nyas.70000

**Published:** 2025-07-22

**Authors:** Piotr Podlipniak

**Affiliations:** ^1^ Department of Musicology Adam Mickiewicz University, ul. Uniwersytetu Poznańskiego7 Poznań Poland

**Keywords:** biomusicology, defense signaling, intraspecific defense, long‐distance calls, musicality, musical pitch

## Abstract

Many species use conspicuous sound signaling as a defense strategy against conspecifics and heterospecifics. This has prompted some scholars to hypothesize that musical displays can serve as a defensive signaling mechanism. It has been suggested that to achieve this function, hominins may have used synchronized acoustic signals, which then became the roots of musical rhythm. Another important component of music—pitch—has been proposed to result either from other selective pressures or as a byproduct of certain biological constraints. This paper aims to add to the idea of the defensive function of music, by looking at the role of pitch matching in the long‐distance deterrence of conspecifics between singing hominins. Since the production and recognition of musical pitch depend on many abilities, their evolution in the context of this possible defensive function is discussed. In particular, the emergence and role of culturally variable musical pitch elements as hallmarks of group identity are examined when considering selective pressures that occurred from increasing hominin social complexity. In contrast to current monofunctional explanations, this proposal highlights a possible reinforcement between other adaptive functions of musical pitch related to the hominin social niche, which ultimately created a synergistic loop and accelerated the evolution of musicality.

## Graphical abstract

The function of pitch matching between hominins as a means of long‐distance deterrence of conspecifics is proposed. The evolution of different abilities related to pitch production and recognition is discussed. In particular, the emergence and role of culturally variable musical pitch elements as hallmarks of group identity are examined, in the context of the selective pressures that occurred from increasing social hominin complexity.

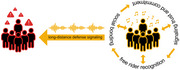

## INTRODUCTION

Pitch is a perceptual sensation that usually accompanies our auditory experience of harmonic sounds. These are complex sounds whose partials (harmonics in this case) are integer multiples of a fundamental frequency (*F*_0_), and they can be produced by both animate and inanimate sources. While humans likely share the ability to recognize harmonic sounds with other primates,^1^ some differences in cortical specialization for pitch processing have been observed between *Homo sapiens* and other primates,^2^ which may be due to the significant role that pitch plays in human‐specific forms of auditory communication. Among them, music is one domain in which the sensation of pitch appears to be especially crucial. Although pitch is also present in almost all forms of human vocalizations such as crying, laughter, and speech, the experience of pitch in music consists of certain additional and specific cognitive properties such as discreteness^3^, ^4^ and circularity.^5^ While pitch discreteness may initially appear to be an objective characteristic of stimuli rather than a cognitive construct, there is ample evidence suggesting otherwise. For example, in the speech‐to‐song transformation,^6^ where looped speech starts to be perceived as a song, the same continuous changes in a vowel's *F*_0_ in speech are interpreted as discrete pitches only because of their repetition and not because of the stability of *F*_0_, which usually differentiates songs and music from speech.^7^, ^8^ This same effect is observed in the sounds‐to‐music transformation.^9^ Such discrete sensations are then incorporated into the circular dimension of pitch, which is characterized in Western music (and many other musical cultures) by the repetition of pitch classes in each octave. While particular discrete pitches are conventionalized, the tendency to discretize pitch in music seems to be a general cross‐cultural phenomenon^10^ which suggests the existence of cognitive constraints related to this feature. Additionally, the experience of pitch in tonal music, which dominates musical cultures globally,^11^ is accompanied by subtle sensations of stability,^12^ resulting in the interpretation of pitch sequences in terms of pitch hierarchies.

The existence of such specific pitch sensations in music leads to the question of their origin. As music appears to be a rather wasteful expenditure, some researchers have proposed that musical pitch is a byproduct of other adaptive abilities such as the language faculty,^13^ auditory scene analysis,^14^ or other nonmusical biological constraints resulting from the features of speech vocalizations.^15^ However, the fact that musical pitch is universal^11^, ^16^, ^17^ and is part of music‐specific mental representations strongly counters any byproduct explanations for its origins. Therefore, many hypotheses indicate possible adaptive values of musicality^18^, ^19^, ^20^, ^21^, ^22^, ^23^, ^24^, ^25^, ^26^, ^27^, ^28^, ^29^, ^30^, ^31^, ^32^, ^33^, ^34^ as a set of abilities that allow the recognition and production of music.^35^, ^36^ Yet, even though the ability to experience musical pitch belongs to one of the crucial components of musicality, most of these hypotheses focus on the adaptive value of musicality in general, and only some look for the specific functions related to particular musical traits.^32^, ^37^, ^38^, ^39^ However, while the origin of musical rhythm that is based on auditory–motor synchronization has often been explained in terms of social bonding^40^ or signaling coalition quality,^20^ musical pitch seems to have received less attention in this respect.

**FIGURE 1:**
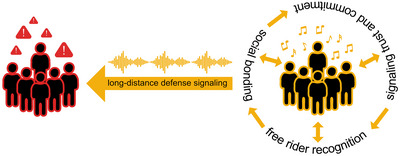
The functions of musical pitch. The same pitch sequence can act as a defense signal or as a tool for social bonding, free rider recognition, and signaling trust and commitment depending on the in‐group or out‐group perspective. *Red*—out‐group hominins; *Yellow*—in‐group hominins.

Admittedly, this topic has recently begun to attract some interest among scholars. It has been suggested, for instance, that chorusing could have become an important part of early hominins’ vocalizations,^41^ serving the social bonding function as a kind of vocal grooming.^42^ Although the evolution of musical pitch might have been related to social consolidation, the vocal grooming hypothesis does not explain why musical pitch has specific features such as discreteness, hierarchy, and so on. Similarly, the idea that musical pitch could have been a fitness indicator as part of a sexual display^26^ leaves the question of the origins of the specific characteristics of musical pitch unanswered. It has been also proposed that the evolution of the melodic part of music originated as a kind of attention signaling in an evolutionary arms race resulting from differences in fitness interests between parents and offspring.^43^, ^44^ Considering that the pitch structure of infant‐directed singing and music^45^ differs from the use of pitch in adult‐directed music,^46^, ^47^, ^48^ this explanation seems at least partially incomplete. In this paper, an alternative view is proposed, in which the pitch part of musicality was shaped by additional selective pressures, including the long‐distance deterrent of conspecific outsiders with malicious intent (see Figure 1). Unlike previous proposals that either neglect the defensive function of music^18^, ^19^, ^24^, ^25^, ^26^, ^29^ or point to the role of rhythm in fulfilling this function,^20^, ^22^, ^34^, ^44^ the hypothesis proposed here emphasizes the importance of pitch as a part of defense signaling. According to this novel view, the adaptive values of musicality do not have to be mutually exclusive,^49^, ^50^ not only because they may be related to different elements of musicality, but also because they may refer to different stages of the evolution of given components of musicality. Therefore, the possible adaptive function of pitch that is proposed here should be considered as an addition to the set of functions such as social bonding,^24^ free rider recognition,^33^ and signaling commitment that may have contributed to the selection of the pitch part of human musicality.

## MUSIC AS A DEFENSE SIGNAL

The idea that music (and dance) could have originated as a hominin defense strategy against predators^21^, ^51^, ^52^, ^53^, ^54^, ^55^ is based on the fact that many species use conspicuous vocalizations as alarm and aposematic signals. In fact, people from some tribal cultures still use musical behavior such as singing and drumming to deter wild animals.^53^ Moreover, some elements of vocalizations can act as cues of aggression, body size, group size, and so forth,^56^, ^57^ which may influence the behavior of predators. It has been observed, for instance, that lions are able to recognize the numerosity of conspecific intruders based on their roaring, which can result in the decision whether to approach them or not.^58^ Although *H. sapiens* is currently the most feared super predator,^59^ which may imply the lack of a need for any defense strategy, in the Early Pleistocene our ancestors were most likely active or confrontational scavengers,^60^, ^61^, ^62^ while not excluding being hunters too,^63^ which involved competition with other predators or scavengers such as lions and giant hyenas for carcasses.^34^ As the size of the hominin group could have given an advantage in this competition,^60^ one of the key factors influencing a lion's or hyena's decision to fight or not for carcasses could have been the cue for the size of the hominin group. Since signals can evolve from cues,^64^, ^65^ the presence of warning vocalizations in primates, which can additionally convey cues of aggression and group size, makes the hypothesis of aposematic signaling as the roots of music likely. Both Hagen and Jordania indicated the main feature of music is most likely to deter these predators is rhythm. This assumption is inferred from observations that many acoustic aposematic signals are synchronized displays, and musical rhythm is based on the human ability to synchronize motor activity, including vocalizations, with auditory stimuli. It is worth mentioning in this context that according to Hagen,^21^ the deterrent function of music does not exclude, but complements the credible signaling hypothesis,^20^, ^44^ which assumes that musical rhythm evolved as a signal of coalition quality (group size and its cooperation ability as well as coalition strength), whereas melody evolved as a signal of parental attention.^44^ By contrast, for Knight and Lewis,^53^ pitch variations could have been used as a defense strategy to deter approaching lions by giving them a misleading sense of hominin group size. Although both musical rhythm and musical pitch are human‐specific signals, which may raise doubts about their recognition by other species, the acquisition of information from heterospecific signals is a common phenomenon in nature^66^, ^67^, ^68^ and can be both inherited^69^ and learned,^70^, ^71^ which makes the defensive hypotheses worth exploring.

## MUSICAL PITCH AS AN INTRASPECIFIC DETERRENT

The origins of hominins are closely linked to the rise of terrestriality, which exposed early humans to greater predation risks and, consequently, intensified selective pressures that favored cooperative defense strategies.^21^, ^72^, ^73^ This collective defense, likely aimed at protecting against large carnivores, would have included the use of stones, sticks, and branches—especially those with sharp thorns^74^—as weapons, alongside physical displays and loud vocalizations.^22^ This strategy would have been further supported by an upright posture linked to the evolution of bipedality, which may have helped scare away predators.^55^ The threat of predation persisted through the emergence of the first *Homo* genus members, suggesting that collective defensive behaviors remained crucial to our ancestors' survival.^75^ Given that both predation pressure and increasing social intricacy are thought to significantly influence communication complexity,^76^, ^77^, ^78^ it seems highly plausible that hominin vocalizations evolved to incorporate the specific use of harmonic sound as an aposematic signal. In this context, protomusical behaviors that functioned as interspecific aposematic signals may have acted as a catalyst for the emergence of protomusical, intraspecific defense signaling, characterized by more complex rhythmic and pitch structures.

Ethnomusicologists have explored for decades the relationship between society and music in terms of their structures and functions.^79^, ^80^ Specifically, they have observed that the size and social composition of a musical group can correlate with specific musical characteristics, as exemplified by the organization of musical ensembles in Pygmy polyphony.^81^ Such a correlation may reflect the proposed causal link between human sociality and the evolution of musicality.^82^ Intergroup competition may have been one of the factors that influenced the tendency of hominins to express social traits through musical structure. After all, when well‐consolidated groups of hominins became a threat, aposematism required more than just the display of numerosity and aggression—it demanded the signaling of coalition strength.^20^ Of course, this scenario does not necessarily exclude the possibility that, in primates, interspecific defense signaling evolved after intraspecific defense signaling. In fact, the need to deter conspecifics has been a significant factor in the evolution not only of hominins but also of other primates as intergroup aggression is a commonly observed behavior.^83^ Although intergroup competition among hominins remains a topic of debate,^84^, ^85^, ^86^, ^87^ with a potential inclination toward intergroup cooperation,^72^ the limited availability of resources could have increased the risk of intergroup conflicts, as is believed to have occurred in the competition between Neanderthals and *H. sapiens* groups.^88^, ^89^

As in the case of predation, the interest of conspecific intruders is to avoid fighting if it is costly and ineffective. Also, signalers producing signals is often less costly than fighting with individuals from a competing group.^90^ Moreover, the ability to receive this information from a long distance allows potential intruders to avoid the risk of entering the territory of a foreign group. Therefore, a credible signal of group strength should also act as an effective defense tool against conspecifics, giving benefits for both senders and receivers of this signal. However, together with increasing social intelligence among hominins, the scope of potentially dangerous interactions between conspecifics would have increased.^91^ As a result, not only group strength, but also other factors such as the strength of bonds between group members, the size and composition of the group, the identity of a formerly met rival, and the group's experience with cooperation and combat would have become important knowledge to acquire that could have influenced the decision to attack or abandon an attack against a specific group.^90^ In such circumstances, what was enough to deter heterospecific predators might not have been sufficient to avoid attack by conspecifics. Moreover, interactions with heterospecific and conspecific outsiders differ in that predators are always a threat, whereas conspecific outsiders can pose both a threat or a benefit resulting from cooperation^90^ (e.g., by being part of a new coalition or as a betrayer of a rival group), which can extend the scope of possible behavioral reactions. All these new challenges could have led to the elaboration of defense strategies including the use of acoustic defense signals.

Although many primates use vocalizations as territorial defense signals which are directed toward conspecifics, these signals are usually instinctive calls^92^, ^93^ sometimes subject to flexibility in use^94^, ^95^ or even characterized by certain developmental plasticity.^96^ In contrast, the most probable important advantage of hominin sound communication that could have been used as defense signaling directed against conspecific intruders was the fact that it was composed of more elaborate culture‐specific parts. In the case of a perpetually changing social environment such as in the composition of groups and the new skills acquired by group members, the culture‐specific elements of signals could have become an important source of information about ongoing changes. After all, such features like a newly invented sound pattern, an improved performance in terms of synchronization and/or pitch matching, and the addition of a new performer, carried an extra set of information to be inferred by conspecifics. It is of course a risk that in the case of the increasing complexity of signals some of the message can become obscured, which can cause a tendency to increase redundancy of the signal.^97^ In fact, music is characterized by immanent repetitions^98^ that may be an effect caused by this risk. As our closest relatives, chimpanzees use both drumming^99^ and vocalization^100^, ^101^ in acoustic signaling, it is therefore reasonable to assume that similar forms of communication were accessible to hominins. However, while the use of percussion tools usually limits produced sounds to rhythmic patterns and makes the sound features dependent on the acoustic properties of percussion instruments,^102^ the use of vocalizations expands the scope of encoded information by adding other spectral features into a means of communication. Expanding the culture‐specific elements of the signaling system on these new possible units based on spectral distinctions (employing timbral and pitch contrasts^103^) increases the combinatoriality of this system. But did the increased complexity of the social system and the new defense challenges it brought contribute to the origins of music? Was the role of pitch in music somehow related to defense signaling?

## A TANGLE OF ULTIMATE EXPLANATIONS FOR THE ORIGINS OF MUSICAL PITCH

Looking for one adaptive function of music seems to be delusive when considering that musicality is composed of many different abilities that did not evolve at the same time. Even the term *musical pitch* may actually refer to a set of biologically meaningful but different signals that may depend on different abilities. One possible way to identify the adaptive functions of the ability to recognize and produce musical pitch is to compare similar abilities and their adaptive functions among species other than humans.^104^ Many abilities that allow for the perception and production of pitch are in fact present in several nonhuman animals.^105^ For example, it has been identified among certain avian^106^ and mammalian^107^ species. Pitch matching has been observed in Japanese macaques (*Macaca fuscata*),^108^ while harmonic intervals have been recognized in the songs of the hermit thrush (*Catharus guttatus*),^109^ the great tit (*Parus major*),^110^ and the musician wren (*Cyphorhinus arada*).^111^ Similarly, the ability to recognize octave equivalence—a sensation of pitch affinity as a result of the perceptive fusion of sounds with a *F*_0_ ratio of 2:1^112^, ^113^—has been observed in dolphins (*Tursiops truncatus*)^114^ and rhesus macaques (*Macaca mulatta*).^115^

Yet, while we share certain features of musicality with other species, none produces music in its full human form.^36^ Likewise, as far as we know, no nonhuman species possess the entire set of abilities mentioned above that characterize the part of musicality that enables the recognition and production of musical pitch. For instance, Wagner and Hoeschele have shown that even the four abilities they identified as crucial for the spectral aspects of human musicality—namely, vocal learning, harmonic clarity in vocalizations, differing vocal ranges, and simultaneous vocalization and duetting—have not been observed together in any other species.^104^ Yet, in order for humans to use musical pitch, it is necessary to have all these abilities together. Although many abilities jointly compose the pitch part of musicality, the origin of each of them, as well as their primary adaptive functions, might not have necessarily been related to musical behavior.^116^ In other words, some of these abilities may have evolved due to the adaptive value of protomusical behavior, while others might have evolved earlier due to different adaptive functions and were only later exapted and incorporated into musicality. For instance, vocal learning could have initially served an important function in broadening the scope of sounds used to communicate propositional meaning.^117^ A good example of such a strategy is the intentional imitation of low frequencies. This strategy could have been an adaptive behavior of communicating a broad meaning related to largeness due to the frequency code^118^—the remnants of which are probably still present in sound symbolism.^119^

Although the volitional vocal control of *F*_0_ may have originally evolved as a tool for protoverbal communication through sound symbols,^117^ it might later have been exapted to intentionally control the pitch aspect of affective prosody. This process could have formed the basis of musical pitch as it took on a new role in deterring conspecifics. It is also possible that the volitional control of *F*_0_ could have been used in order to communicate other meanings such as dominance, strength, masculinity, or femininity, depending on the social context.^120^ A similarly intricate evolutionary path could have led to the emergence of the other abilities connected with musical pitch.^24^ However, taking into account the variety of physiological mechanisms involved in the production and processing of musical pitch,^121^, ^122^, ^123^, ^124^, ^125^ the evolution of each of them as the result of one adaptive function seems to be unlikely. It is also possible that the convergent or parallel evolution of some abilities could have occurred in response to different selective pressures.^126^ Therefore, octave equivalence, for instance, could have evolved independently in dolphins and primates as a tool to enhance communication by means of harmonic sounds, but in response to different functions of such communication.

Similarities between sounds composing octave could be used to restrict the number of units in a communicative system, or could have just been used for the expression of similarity in an iconic way. It is therefore presumable that octave equivalence among *H*. *sapiens* evolved due to additional selective pressures, for example, those related to social functions.^127^ This does not exclude the possibility that the tendency to recognize pitches that share many overtones as similar may be a trait that evolved long before hominins.^104^ Sexual dimorphism in voice pitch among humans, which amounts to approximately one octave, is significantly greater than that observed in other apes.^128^ The hypothesis that sexual selection alone drove the evolution of octave equivalence appears to be insufficient,^127^ particularly given that the *F*_0_ difference between male and female voices typically diminishes with the transition to monogamy.^128^ Since monogamy is the predominant mating system in *H*. *sapiens*, such a pronounced disparity in voice pitch between men and women likely stems from additional selective pressures. For instance, the act of male and female hominins singing together in octaves may have conferred an advantage in long‐distance group signaling (see the section “The role of timbre, pitch, heterophony, and vibrato in signaling group size”). Thus, while the initial emergence of voice dimorphism in our ancestors was most likely driven by sexual selection,^129^ the pitch difference between men and women eventually became a constraint influencing the evolution of vocal defense signaling. To achieve pitch matching and overcome this constraint, singing in octaves may have proved to be the most effective solution.

The intraspecific defensive function of musical pitch mentioned above can be attributed to the use of discrete pitch patterns and the evolution of sensitivity to them.^30^ From this perspective, pitch matching between singers can act as a credible signal of group size and cohesion. Such a signal decoded by the in‐group members probably served a different function than that detected by out‐group members, thus being attractive for the former and aversive for the latter. After all, the same signal can cause different reactions depending on its usefulness for a given individual. For example, the distress signal of a baby crying motivates caregivers to care,^130^, ^131^ while it encourages crocodiles to prowl for easy prey.^132^

A similar effect could have impacted the evolution of musical pitch among conspecifics. Different context‐based meanings of the same signal (submission and subordination) have been observed in primates,^133^ which makes it likely that a similar mechanism may have occurred in hominins using pitch matching as a form of collective vocalization. The defensive function of pitch patterns could have then been enhanced by a cultural variability of these signals as the emblems of particular groups, becoming at the same time a kind of friend or foe identification system. Such a strategy, often named password signaling, has been observed in many vocal learners such as cetaceans^134^, ^135^ and birds.^136^, ^137^, ^138^ Fitch has hypothesized that password signaling could have been a reason for the evolution of human vocal learning.^139^ According to Fitch, the human ability to recognize natives via a language accent speaks to this hypothesis. However, when taking into account that the recognition of a foreign accent is mainly influenced by speech rhythm,^140^ which is based on amplitude modulation,^141^ password signaling by means of speech seems to be restricted to short distances (see the next section). In contrast, the changes of *F*_0_ that are crucial for the recognition of pitch seems to be a better choice as a means to advertise group belonging over long distances. In noisy conditions, vowels—being harmonic sounds—are more easily recognized over long distances than consonants.^142^ The advantage of harmonic sounds in long‐distance communication is further supported by the fact that many cultures use whistle languages, composed entirely of harmonic sounds, as tools for such communication.^143^

Hagen and Hammerstein have suggested that musical variability could have evolved due to the need for communal identity signaling as part of territorial advertisement.^34^ They have also emphasized the crucial role of vocal learning in this task. Therefore, the implicit learning of a culture‐specific musical pitch system by contemporary humans may be a canalized remnant of using musical pitch as a defense signal. Above all, what is an easy task for us today could have been a greater challenge for our ancestors. For hominins with restricted vocal learning, adjusting to any pitch pattern must have been a strenuous task not only because of the demands on memory, but also because of the need to be in tune with a particular group‐specific tuning. Being time‐consuming and strenuous on the one hand, and adaptive as a signal of group consolidation that defends against hostile out‐group conspecific attacks on the other, this sensitivity to tuning could have become an instinctive learning strategy. Instinctive learning, being a type of learning based on innate learning preferences which guide and facilitate the learning process,^144^ may have given an advantage to individuals by confirming belonging and commitment to the group as well as deterring out‐group hostile conspecifics. After this evolution of sensitivity to tuning, the process of an arms race of learning abilities between individuals may have continued.^33^ The new challenge, however, was pitch distribution.^32^ In both cases of sensitivity to tuning and sensitivity to pitch distribution, the experience of well‐matched pitches could have caused positive emotional reactions in group members singing together. In contrast, such well‐matched patterns could have triggered awe and fear in out‐group conspecifics, serving as an effective defense strategy.

## HARMONIC SOUNDS AS DEFENSE SIGNALS IN NONHUMAN TERRESTRIAL ANIMALS

As fighting is often more costly than sound production, animals often use sounds as defense signals aimed at both conspecifics and heterospecifics.^90^, ^145^ Some of these behaviors can be very conservative, whereas others can evolve independently. As convergent evolution provides a special opportunity to recognize common ecological conditions that could have contributed to the evolution of similar traits among species not closely related,^146^ it is useful to look at the defense signals used by different terrestrial animals. After all, similar defense signals used by unrelated species could have been caused by similar selective pressures. Many examples of defensive sound signals consist of broadband sounds. Energy is distributed over a large range of the spectrum in growling by the domestic cat (*Felis silvestris catus*),^147^ dingo (*Canis lupus dingo*),^148^ domestic dog (*Canis familiaris*),^149^ Indian wolf (*Canis lupus pallipes*),^150^ and so forth. Also, hissing by the domestic goose,^151^ blue tit (*Cyanistes caeruleus*),^152^ domestic cat (*F*. *silvestris catus*),^147^ puff adder (*Bitis arietans*),^153^ bumblebee (*Bombus terrestris*),^154^ and Asian dwarf honeybee (*Apis florea*)^155^ consists of such sounds. In fact, broadband sounds have many advantages in respect to defense functions. First, the wide distribution of energy on the partials of different frequencies provides enough redundancy that would be hard to ignore by the recipient. Second, broadband sounds are difficult to be masked entirely by environmental sounds. Finally, such a wide distribution of acoustic energy makes the signal accessible to various species that have different auditory frequency ranges. However, if a signal is to act over a long distance, its effectiveness depends on its robustness of sound propagation in the air. In this respect, broadband sounds are quite poor as they are susceptible to scattering and attenuation.^156^

**FIGURE 2:**
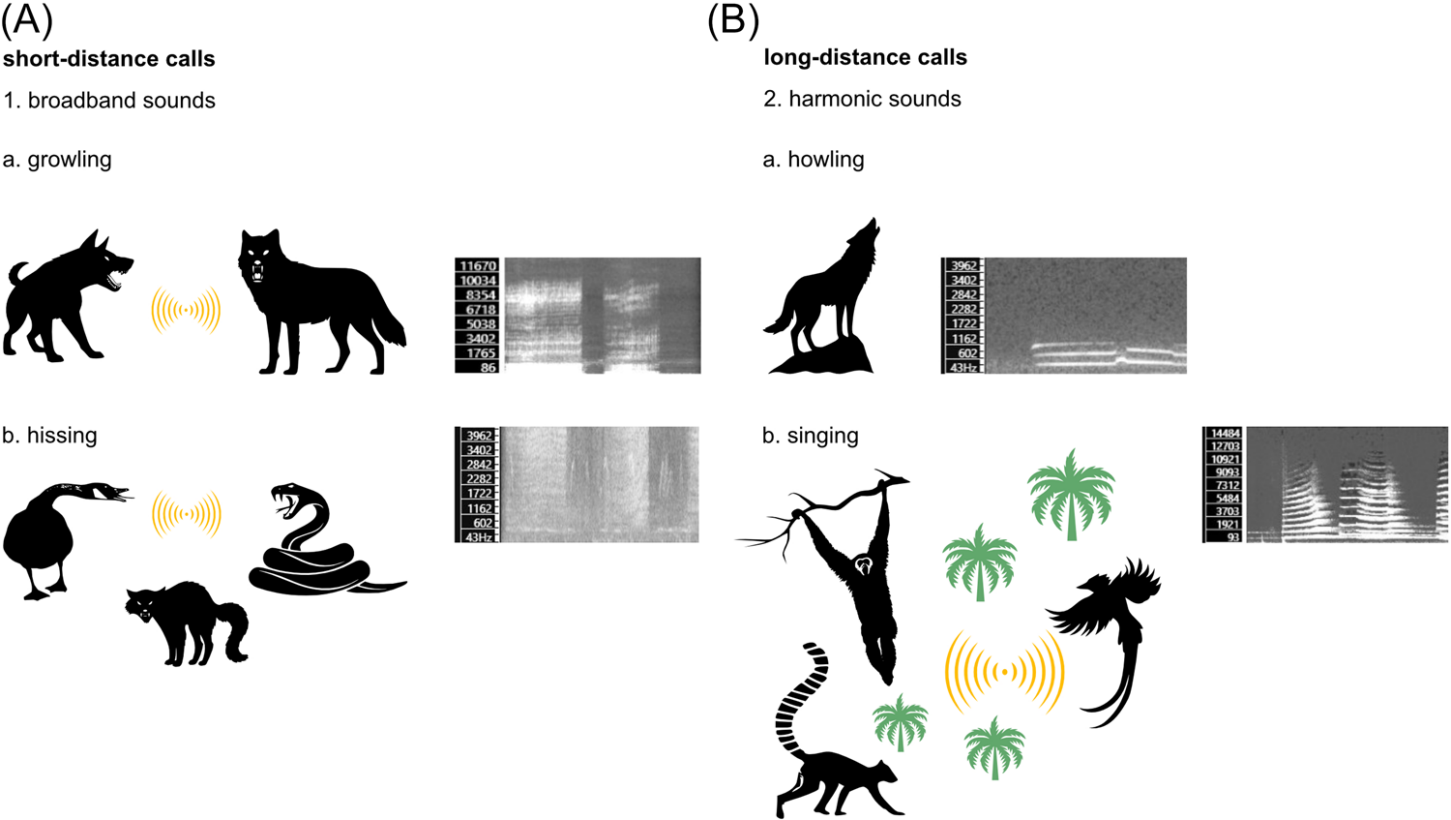
The two types of vocal signaling: (A) short‐distance calls: a—growling (the spectrogram of a dog growl), b—hissing (the spectrogram of a hissing goose); (B) long‐distance calls: a—howling (the spectrogram of a wolf's howl), b—singing (the spectrogram of an indri song).

In contrast, harmonic sounds, especially at low frequencies, are much more effective at propagation in the air at longer distances.^157^, ^158^, ^159^ Therefore, terrestrial animals usually use harmonic sounds in sound communication whenever the potential recipients of the signals are located at a large distance (see Figure 2). This observation led to the development of the acoustic adaptation hypothesis, which posits that long‐distance vocalizations evolve and persist because they are optimally transmitted within the specific habitats animals inhabit.^160^ One prediction of this hypothesis is that in closed habitats, vocalizations tend to be longer, lower in frequency, and less repetitive, whereas in open habitats, the opposite pattern is expected. Although a computer simulation has supported the validity of this hypothesis,^161^ more recent meta‐analyses of empirical studies on amphibians, birds, and mammals have not provided consistent support.^162^

Nevertheless, the use of harmonic sounds in long‐distance signaling is often observed among terrestrial animals. This rule seems to apply to defense signals as well. Of course, harmonicity is not the sole factor influencing the effectiveness of signal propagation, with sound intensity and background noise^163^ ranking among the most significant. Therefore, the use of harmonic sounds in this type of signaling cannot be regarded as an absolute rule. A need to deter intruders from long distances is especially important in territorial species.^164^, ^165^ After all, in order to sustain exclusive access to resources such as shelter, food, and mates, it is crucial to determine and mark the boundaries of the inhabited territory.^166^, ^167^, ^168^ According to Hagen and Hammerstein, the most common form of acoustic territorial advertisement is vocalization.^34^ In fact, vocalizations composed of harmonic sounds are used as a territorial defense by many species of birds^169^, ^170^, ^171^ and mammals.^150^, ^172^, ^173^, ^174^, ^175^, ^176^, ^177^ Interestingly, harmonic sounds are also used in emergency sirens, which has led to the speculation about the possible role of the human predisposition to an attentional response to howling wolves and the invention of the siren.^178^ But this response can be the result of an evolutionarily older sensitivity to loud harmonic sounds. Early species of the *Homo* genus were probably cooperative hunters,^179^ which makes them similar to wolves, lions, and hyenas in that respect. Since all these animals advertise their territory by means of loud pitched vocalizations such as howling, roaring, and whooping, respectively, one would suspect that hominins also used defense signaling that could have resembled elements of these vocalizations.^34^

Among mammals that use harmonic sounds in their long‐distance calls, there are also primates such as marmosets,^180^, ^181^, ^182^ tamarins,^182^, ^183^ titi monkeys,^184^, ^185^ langurs,^186^ indris,^187^ tarsiers,^188^ and gibbons.^28^, ^189^ The ubiquity of long‐distance vocalizations among primates suggests that human musicality has its roots in this behavior.^28^ A common form of vocalization observed among these primates is duetting. In exploring the evolution of human musicality, researchers have emphasized the role of synchronization^190^, ^191^ and pair bonding^28^ in relation to primate duetting. It is worth mentioning, however, that minimal volitional control of pitch has been observed among many primates,^192^, ^193^, ^194^ including gibbons,^195^ which opens the door to the possibility that some elements of vocal learning related to human singing could have been inherited from the common arboreal ancestor of humans and apes.^54^, ^55^ Although the correlation between primate duetting and monogamy has been interpreted as a strong indication for the role of sexual selection in the evolution of primate singing,^186^, ^190^ it should not be ignored that primate songs are also used as territorial defense signals,^185^, ^196^, ^197^ which speaks for the defense hypothesis of music origin. It should also not be ignored that harmonic sounds are an important element of these vocalizations, which prompts us to consider the role of pitch as an important factor in our hominin ancestor's defensive signaling over long distances, even though some primates, such as chimpanzees (*Pan troglodytes*), do not sing.^198^

Lack of singing does not mean, however, that chimps do not use harmonic sounds in their vocalizations. Indeed, they are present in many types of chimpanzee vocalizations such as in laughter, screams, hoo calls, pant‐hoots, barks, squeaks, as well as in some types of grunts and whimpers.^101^ Harmonic sounds are also an important component of affective prosody,^199^ elements of which are present in the vocalizations of many mammalian species,^200^, ^201^ including the aforementioned vocalizations of chimpanzees. For many scholars affective prosody seems to be a good candidate to be a direct precursor of both speech and singing in humans.^31^, ^202^, ^203^, ^204^, ^205^, ^206^ Perhaps affective prosody also gave rise to the most complex vocalization specific to chimpanzees and bonobos (*Pan paniscus*), that is, pant‐hooting—a form of long‐distance communication characterized by a sequence of hoo calls and screams.^101^ Pant‐hooting can also be occasionally combined with rough grunts, which suggests volitional control over its production,^207^ and seems to develop later in ontogenesis in comparison to other types of chimp calls.^101^ Although territorial defense is not the only function proposed for pant‐hooting,^208^ the fact that social functions such as signaling social bonds^209^ or social status,^210^ attracting conspecifics to food,^211^ and facilitating fusion,^212^ have been indicated among its many different functions and suggests that chimpanzee vocalizations are functionally similar to those of other primates. This means that long‐distance hominin calls could have also been under selective pressure related to conspecific social environments. An important difference between chimpanzee and *H*. *sapiens* long‐distance vocalizations, which can be a result of this selective pressure, is the more elaborate cultural variability of the latter.

## THE AFFILIATIVE VERSUS INTIMIDATING IMPACT OF PITCH

Harmonic sounds are usually used by many species in affiliative contexts such as mating and other positive social interactions that often lead to increased social cohesion. Many species of birds, for instance, sing to advertise sexually,^213^, ^214^ to maintain social bonds,^215^ to coordinate behavior, and to facilitate complex social interactions.^216^ Also, many species of mammals,^217^ including primates,^218^, ^219^ produce harmonic sounds in similar contexts. However, each vocal message in addition to affecting the selected recipient is exposed to eavesdropping. This threat concerns both the risk of being noticed by a predator^132^, ^220^, ^221^ and by a conspecific^222^ competing for various resources with the sender of the message. As far as the risk of predation is concerned, a blending effect caused by pitch matching can make it difficult to recognize or trace a particular individual by a predator. At the same time amplitude summation of sounds produced during chorusing can contribute to the perceived body size exaggeration effect. In social species, if the risk of eavesdropping by conspecifics is high, natural selection can favor such affiliative signals which additionally contain a warning–threat message that encodes information about coalition strength or group cohesion, for example. In many cases of warning signals, including those that operate over long distances, it is the rhythm not the melody that seems to be primarily related to the deterrent function.^223^ A notable example of defensive signals where rhythm plays a crucial role is barking, which can be detected from long distances. In addition to being composed of rhythmic, noisy, and repetitive abrupt vocalizations, barking also often includes harmonic sounds.^224^ Although barking is usually associated with domestic dogs similar mobbing calls have been observed in many other taxa, such as rodents,^225^ birds,^226^ and primates.^227^ In fact, the exposure of primates to danger such as predators or intruders often involves repetitious and rhythmic calls,^228^ often similar to barking.^163^, ^227^, ^229^

In many social species, including primates, rhythmic calls serve as long‐distance territorial signals. For instance, it has been observed that white‐handed gibbons (*Hylobates lar*) use songs to deter intruding conspecifics.^189^, ^230^ Although auditory–motor synchronization, a key component of human musicality, has so far been observed in chimpanzees only in a rudimentary form,^231^, ^232^ the vocalizations of primates such as lemurs,^233^ gibbons,^234^, ^235^ and orangutans^236^ are characterized by isochrony or other types of categorical rhythms. Rhythm is also a particularly important feature of chimpanzee drumming,^237^ which is thought to be a specific type of agonistic display.^238^, ^239^ Importantly, chimpanzee drumming is often accompanied by vocalizations that are also isochronous.^237^ All these observations suggest that the basic cognitive mechanisms that underly the rhythmic part of human musicality were shared by our last common ancestor with chimpanzees. Moreover, the use of coordinated drumming by chimpanzees to intimidate conspecifics indicates that rhythm was probably a crucial component of hominin defensive signaling. For Hagen and Bryant the use of rhythm by hominins as a part of coalition signaling display had a precursor in such rhythmic territorial defense signals.^20^ Also Fitch and Zuberbühler have proposed that defensive displays against predators could have been an adaptive function for hominin rhythmic vocal calls.^22^ They have emphasized that the effectiveness of synchronized group signaling is due to the acoustic summation of amplitudes, which increases signal loudness.

As synchrony necessitates coordination, it is a good marker of coalition strength which can both intimidate enemies and encourage cooperation.^20^ Nevertheless, if the same signal is to fulfill two different functions, depending on the “friend or foe” perspective, pitch can also act as a ritualized tool of intimidation, especially if it is well matched and additionally well synchronized. In this context, the hypothesis that the human ability to perceive and synchronize to a beat may have evolved as a byproduct of vocal production learning,^240^ can be reversed. Synchronized displays may have emerged among Australopithecines—or even earlier—as a mechanism to deter predators^22^ or to signal dominance and cohesion among conspecifics.^20^

**FIGURE 3:**
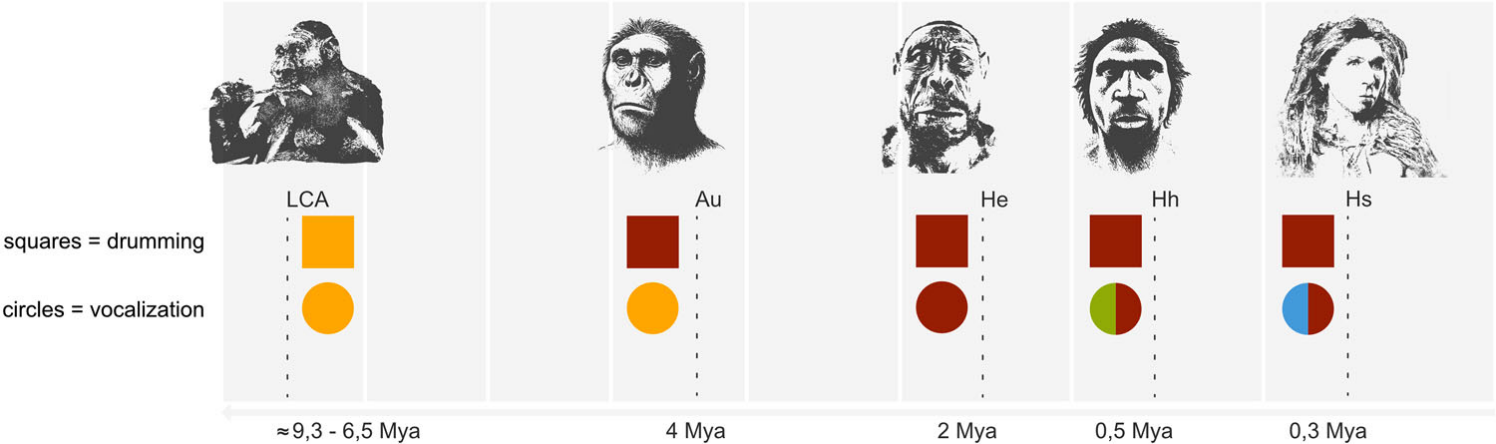
The evolution of vocal defense signaling in the *Homo sapiens* lineage: Yellow, isochrony/asynchrony; red, synchrony; green, heterophony; blue, pitch matching. Au, Australopithecines; He, *Homo erectus*; Hh, *Homo heidelbergensis*; Hs, *Homo sapiens*; LCA, last common ancestor of chimpanzees and humans.

Given this evolutionary context, it is plausible that the capacity to perceive and synchronize to a beat functioned as a preadaptation for vocal production learning in hominins. An important factor that may have additionally influenced the evolution of the ability to perceive and synchronize to a beat was the transition to bipedalism^31^, ^241^ and endurance locomotion.^242^ Given that human vocal production learning is a complex ability composed of multiple elements^243^, ^244^, ^245^, ^246^ that likely evolved gradually over time, the imitation of the temporal order of sounds alone might have been one of the earliest components of vocal production learning in hominins. The fundamental role of rhythm in defense signaling suggests that the evolution of vocal learning in our lineage may have started exactly with the temporal control of vocalizations in the predecessors of the *Homo* genus, probably even in *Ardipithecus ramidus*.^241^, ^247^ This development ultimately enhanced the vocal control of *F*_0_ in *Homo erectus* and their descendants (see Figure 3).^241^ Apart from this, synchronization can be viewed as a rhythm analogue of pitch matching as synchronization of sounds is in fact a kind of alignment and can act as a signal of cohesion.^20^ This simplified form of alignment, compared to pitch matching, might have been inadequate in deterring conspecific intruders if the arms race among hominins resulted in the widespread adoption of this tool as a defense strategy. After all, synchronization, being time‐dependent,^248^ is inherently limited in complexity. Incorporating pitch matching into a synchronized display adds an extra dimension to signaling, thereby enriching the information conveyed.

In many cultures such displays are accompanied by rhythmic entrainment,^249^ which is not only an analogous form of pitch matching, but also appears to be a key mechanism for sustaining prolonged performance. In this context, the duration of the performance may serve as a significant indicator of stamina, further contributing to deterring conspecific intruders. From this perspective, rhythm is not a defining feature of short‐distance defense signals but rather one of the signaling tools that can be enhanced by pitch. Specifically, rhythmic sequences of broadband sounds are less effective in propagation compared to rhythmic harmonic sounds, such as the duets of siamang^250^ and tarsier.^251^ In humans, the combination of rhythm synchronization and pitch matching results in sequence alignment, which appears to be a powerful tool for exerting social influence over long distances.

Nowadays, musical behavior is used in many rituals in which the meaning is often related to combat, intimidation, and threat display,^252^ as in the case of the Māori *haka* performance,^253^ football fan chants and songs,^254^ and in military songs.^255^, ^256^, ^257^ Lehmann et al. have indicated 20 ethnological examples of dual songs such as Inuit *piseq* songs, *tiv* songs from Nigeria, and musical territorial negotiations by Yanomami people from Venezuela.^258^ While musical duels represent a kind of competition between soloists, the use of ritualized, musicalized speech by competitors may reflect a need to emphasize the verbal message via an additional intimidating signal. Although, according to Lehmann et al., the ubiquity of this tradition can be a result of intrasexual selection, the presence of similar intimidating functions in the use of military and football fan songs may suggest that this function of music could have evolved due to selection by hominin social environments. Both in military songs and football fan chants, the message encoded by words seems to be less important than the influence of musical structure. This suggests that the intimidating power of music has its roots in preverbal collective rituals rather than being a product of intrasexual selection.

## THE ROLE OF TIMBRE, PITCH, HETEROPHONY, AND VIBRATO IN SIGNALING GROUP SIZE

The size of a group plays a crucial role in determining its defensive effectiveness.^259^ Before launching an attack predators and conspecific intruders typically assess their likelihood of success, and a large group of defenders can serve as a formidable barrier—or even pose a counter‐threat. As a result, accurately estimating group size is essential for intruders, while defenders may benefit from exaggerating their numbers. Additionally, the ability to signal group size over long distances can be advantageous as it may deter potential intruders from approaching the territory. In order to distinguish between different individuals by their voices, each voice must be processed in the auditory system as a separate auditory stream.^260^ Timbre and *F*_0_ are typically effective cues for auditory stream segregation.^261^, ^262^, ^263^ However, when simultaneous voices are similar in *F*_0_ and timbre, separating them into distinct auditory streams becomes challenging, leading to the loss of information about the exact number of individuals. A potential solution to this issue may have been the use of varied timbres and pitches by individual hominins. This strategy bears resemblance to the chorusing behavior of howler monkeys whose loud roars are often used to repel opponents from rival groups.^264^ The distance over which these vocalizations can be heard often matches or even exceeds that of chimpanzee pant‐hoots.^163^ Nevertheless, in such cases, the range of audibility and recognition of the message, especially roars and barks,^265^ is limited^266^ compared to the solo barks or duets of gibbons^267^ or the group singing of humans in unison or octaves.

These acoustic constraints seem to require a trade‐off between signaling group size and signal range. When a group consists of around a dozen individuals, its size can be estimated through auditory stream segregation. Certain musical features—such as varied timbres, vibrato, polyphony, and heterophony—undoubtedly facilitate this process. The idea that the evolutionary precursor to music was based on heterophony characterized by poor synchronization^268^ and pitch matching^203^, ^269^ is consistent with this function. However, since the counting abilities of mammalian predators and hominin intruders were limited,^270^ in cases where a hominin group consisted of several dozen individuals, the estimation of group size likely relied upon approximation rather than precise counting of individuals. In such a case, it seems to be a better solution to signal the group size using sound intensity. As synchronized and pitch‐matched signals increase sound intensity through amplitude summation, group singing in unison or in octaves may have served as an honest signal not only of group size but also of cooperativity.^27^

In this respect, an important point not to be overlooked is the collective singing of men and women who can match pitches by singing in octaves. In fact, even if male and female voices differ in timbre those that share the same pitch are often difficult to distinguish.^104^ However, the vocal intensity of individual singers may vary depending on their body size and vocal capacity, which makes is more difficult to accurately estimate group size. This could have opened the door to deceptive strategies, which may have in turn created selective pressure for enhanced abilities in synchronization, pitch matching, and vocal intensity. In this respect, group singing—unlike other forms of human vocalization—can operate on a much larger scale and bring together dozens of individuals.^127^ Consequently, as group size increased, the tendency to diversify individual vocalizations in terms of timbre and pitch may have given way to selective pressure for pitch matching. This growth in group size may have also contributed to the evolution of octave equivalence, as the blending of male and female voices amplifies loudness, effectively signaling the group's size and deterring conspecific intruders by emphasizing group unity. It is also possible that the harmonic clarity that distinguishes *H*. *sapiens* vocalizations from those of many other primates^104^ is a result of this evolutionary process.

## VOCAL PRODUCTION LEARNING AS A PRECONDITION FOR MUSICAL PITCH

As the musical use of pitch in hominin history probably started with vocalizations,^102^, ^271^ one can assume that the evolution of the pitch part of human musicality must have been related to singing. To sing culturally variable songs, the voluntary laryngeal control of *F*_0_ had to evolve.^271^ This capacity is a part of the broader ability called vocal production learning, which distinguishes *H*. *sapiens* from other living primates^272^, ^273^ including chimpanzees, meaning that it had to evolve in our evolutionary lineage after the split from our last common ancestor with chimpanzees. However, it has been observed in some species of birds^274^ and mammals.^245^, ^272^ Interestingly, some of these vocal learning species such as the red‐winged blackbird (*Agelaius phoeniceus*),^275^ the yellow‐breasted boubous (*Laniarius atroflavus*),^276^ the banded wren (*Thryophilus pleurostictus*),^164^ and the great Himalayan leaf‐nosed bat (*Hipposideros armiger*)^176^, ^177^ use their vocalizations as territorial defense signals, which implies that apart from sexual display and information sharing,^277^ one should also take into account a defensive function as a potential reason for the evolution of vocal learning among hominins. Moreover, considering the flexibility of use and developmental plasticity of primate calls,^95^, ^96^ the gradual evolution of vocal learning may have even begun in the last common ancestors of humans and gibbons. It is worth emphasizing that developmental learning among primates continues into adulthood,^278^ influencing the plasticity of vocal development in primates^279^ including chimpanzees.^280^ Although in general vocal production learning is defined as an ability to reproduce by voice what is heard,^281^ vocal learners differ in their capability to reproduce sounds, meaning that vocal learning can be seen as a continuum of abilities rather than a yes or no ability.^243^ Moreover, these differences could concern various parameters such as the accuracy of the copy, the degree of possible modifications, and what is being copied.^282^ It can be assumed that different adaptive functions that result in structurally different vocalizations should impose separate and specific characteristics on species‐specific vocal learning. This difference is evident in the case of human vocally learned forms of communication such as speech and song.

**FIGURE 4:**
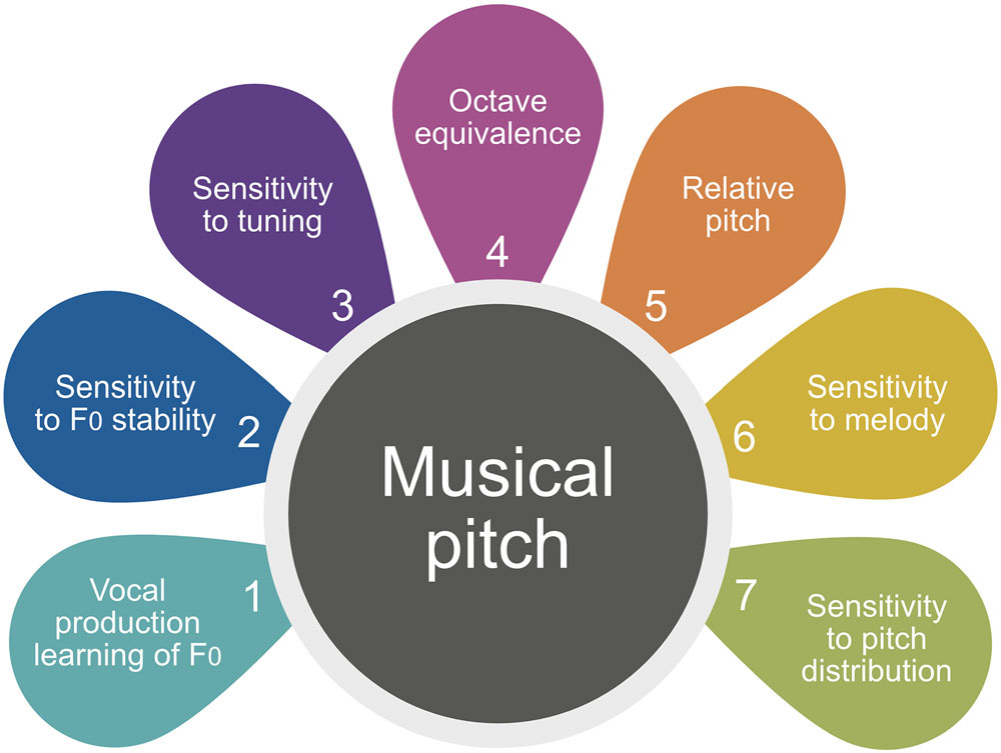
Music‐specific abilities related to pitch. Vocal production learning of *F*_0_, allows hominins to volitionally control and sustain *F*_0_ and align their vocalized pitch with other singers; Sensitivity to *F*_0_ stability, enables hominins to interpret harmonic sounds in terms of discrete pitch intervals; Sensitivity to tuning, enables the recognition of a culture‐specific pitch system; Octave equivalence, restricts the scope of a pitch system; Relative pitch, enables hominins to recognize transposed melodies as the same pitch patterns; Sensitivity to melody, facilitates the veridical memory of vocalized melodies; Sensitivity to pitch distribution, enables the interpretation of pitch sequences in terms of tonal hierarchy.

As the role of the temporal envelope and temporal fine structure differs in the perception of speech and music^283^, ^284^, ^285^, ^286^ one can suspect that vocal production learning of these two communicative forms is also based, at least partly, on the imitation of different acoustic features. This difference seems to be evident at the cognitive level since the recognition of musical structure is grounded in pitch and rhythm,^4^, ^287^, ^288^ whereas speech is perceived mainly as a chain of changing vowels and consonants that are recognizable due to the contrast of formants.^289^ Even the rhythms of speech and music, encoded through modulations of sound intensity over time differ and exhibit well‐separated peaks—around 5 Hz for speech and 2 Hz for music.^290^ Consequently, the imitation of these two vocal expressions likely depends on distinct rhythmic biases. Since music differs from speech in its much higher degree of repetition,^291^ recognizing these repetitions may also play an important role in distinguishing music from speech. The use of repetitive musical phrases, though likely rooted in more general abilities to recognize similar patterns, also requires vocal production learning of pitch. The crucial part of human vocal production learning that determines the use of pitch in music is the volitional control of *F*_0_. In fact, it is this ability that allows us to imitate the pitches we hear.^292^ While pitch is also an important part of speech and can imply that the volitional control of *F*_0_ evolved as a part of the language faculty,^293^ both the stability of *F*_0_ ^7^, ^8^ and its syntactic function^294^ seem to be much more important in music than in speech. Thus, the ability to sustain *F*_0_ as a part of vocal production learning must have been selected for because of the adaptive functions that formed the basis of musicality and not the speech faculty. If the stability of *F*_0_ was such an important acoustic feature of hominin protomusic, then it would have also become an indicator for music recognition and comprehension. This means that the volitional control of *F*_0_ must have coevolved with a perceptive sensitivity to pitch stability (see Figure 4).

## PITCH DISCRETENESS, COMBINATORIALITY, AND LONG‐DISTANCE CALLS

The recognition and vocalization of sounds with different stable *F*_0_ must have also become the basis for another important feature of musical pitch discreteness (see Figure 4). Only hominins endowed with these two abilities could have vocalized pitches in a discrete‐like fashion—also in relation to discrete units of time—that characterize contemporary singing and music.^4^ In other words, the emergence of pitch discreteness was related to the natural selection of the volitional control and sustenance of *F*_0_ as well as sensitivity to pitch stability. This is not to suggest that the ability to produce a stable *F*_0_ is an exceptional skill exclusive to humans. However, the difference between the stability of harmonic sounds in speech and in music may reflect the difference between the ranges of information transfer achieved by these two forms of communication. While speech is used to exchange information over a short distance, singing allows a signal to be transmitted over a longer distance. Since reverberations of soundwaves impede the intelligibility of calls characterized by rapid frequency modulations,^156^ the use of stable *F*_0_ can be one of the factors that facilitate long‐distance communication, especially in a forested environment that causes many soundwave reverberations. This means that in such an environment, in order to send a message efficiently over a long distance it is better to code information in an acoustic signal by means of frequency rather than amplitude modulation. Although *Homo* species have been thought to live in open areas,^295^ some studies suggest that their habitats were much more diverse^296^, ^297^ including forested landscapes.^298^ Thus, the ability to vocally control *F*_0_ stability in singing could have evolved as an adaptation to long‐distance vocal signaling.

Musical pitch is also important for the combinatoriality of music (see Figure 4).^299^, ^300^ Music is claimed to be an example of the Humboldt system^4^ (i.e., a system which is based on a restricted number of discrete units combined according to certain rules). The big advantage of such a system is its power to generate an enormous number of expressions from a finite number of units.^301^ An important feature of our experience of harmonic sounds that restricts the number of pitches (units) is octave equivalence (see Figure 4).^5^ Due to octave equivalence, the whole range of audible sound frequencies is divided into equivalent parts—octaves which create a circularity of pitch space.^5^, ^302^ Since octave‐based pitch space appears to dominate musical cultures,^17^ and the ability to perceive octave equivalence seems independent of speech abilities^303^—despite influencing speech^304^—it should be considered a fundamental aspect of the pitch component of musicality. That is not to say that octave equivalence is specific solely to music and that it evolved because of selective pressures related to musicality. Since it has been observed in nonhuman species,^114^, ^115^ the use of this ability in musical behavior should rather be considered an effect of repurposing a pre‐existing cognitive tool.^305^, ^306^ Independent of whether the ubiquity of octave equivalence in musical cultures is an effect of convergent cultural evolution,^307^ or a human‐specific trait based on a perception‐production feedback loop,^127^ the widespread presence of octave in musical pitch systems indicates its importance for musicality. Although some scholars have questioned the universality of octave equivalence,^308^ relative pitch—the ability to recognize two‐pitch patterns as the same melody if their *F*_0_ follow logarithmic relationships (i.e., based on the same frequency ratios)—appears to be independent of cultural background.^309^ Thanks to relative pitch the identity of transposed melodies is preserved.

## SENSITIVEY TO TUNING, PITCH MATCHING, AND THE TWO TYPES OF MEMORIES FOR PITCHES

An important consequence of pitch discreteness and circularity is the emergence of a musical pitch system. While the discreteness of musical pitch is a universal feature of music,^10^, ^17^, ^310^, ^311^ musical pitch systems vary cross‐culturally both in terms of the number of pitch classes and the size of pitch intervals.^312^ This means that musical pitch systems are culture‐specific. Nevertheless, sensitivity to any musical pitch system starts very early in ontogenesis,^313^, ^314^ which suggests that sensitivity to tuning is based on so‐called experience–expectant plasticity.^315^ This type of plasticity implies a genetically constrained developmental path^116^ suggesting that sensitivity to a musical pitch system could have also been selected as a part of our musicality.

However, although humans are quite good at the discrimination of even small differences in the *F*_0_ of harmonic sounds,^316^ even in a musical context^317^ the recognition of pitch intervals allows for greater or lesser tolerance.^3^, ^318^, ^319^ Admittedly, the tuning of pitch intervals by singing individuals is usually less precise^320^ than one would expect from the human ability of *F*_0_ discrimination. Nevertheless, the observed tendency of singing individuals toward intonation accuracy^321^ indicates that sensitivity to tuning is not only a passive, but also an active part of human musicality (see Figure 4). Therefore, the consequence of active sensitivity to tuning is the ability to match pitches among individuals, not only the production of sounds in general but the selected spectral characteristics of these sounds such as *F*_0_ and other harmonics.

The effect of collective singing based on the sensitivity to tuning can be called pitch matching among singing individuals. Aligning pitches not only imposes additional demands on working memory for pitch but also entails the special role of predictions. After all, to sing a particular melody together, singers need to know the pitches of which it consists. Contemporary humans are assumed to be endowed with two types of musical expectations—schematic and veridical.^322^ While schematic expectations in the domain of pitch require sensitivity to pitch distribution, veridical expectations demand sensitivity to specific melodic patterns.

Pitch distribution has been shown to be one of the main foundations for pitch syntax—tonality^12^, ^323^, ^324^—which has often been claimed to be an additional dimension of musical pitch (see Figure 4).^325^ This dimension, which is described as a hierarchy of pitches, is based on subtle sensations—tonal qualia^12^—that accompany the experience of musical pitch in tonal music. For some researchers, this hierarchy is merely a consequence of the harmonic structure of periodic sounds, as they explore, for example, the origins of pitch centers in functional consonance, which arises from the simple relationships between harmonics of particular pitches.^326^ In contrast to acoustic explanations, many cognitive mechanisms such as the exposure effect,^323^ prediction effect,^12^ and physiological nonlinearities in the auditory pathway^327^, ^328^ have been proposed to explain tonal hierarchy. All these proposals refer to the general cognitive mechanisms which may suggest that tonality is not based on music‐specific abilities. From this perspective, the emergence of tonality resulted from a solely cultural transmission process.^146^

However, from a behavioral point of view, tonality (in the sense of a psychological hierarchy of pitches based on tonal center) is a specific psychological trait unique solely to music, which is a challenge for any explanation that refers to general cognitive mechanisms. One can imagine that purely cultural intergenerational transmission of musical patterns was enough for the emergence of tonality by means of a learner bottleneck.^329^ According to this explanation, tonal hierarchy should have become part of musical culture without any external cause or function needed to do so. In this case, sensitivity to pitch distribution is based on another type of plasticity—experience‐dependent plasticity.^315^ A possible alternative to the purely cultural origin of tonality is the canalization of a specific integration of many cognitive mechanisms that arose as a phenotypic adaptation in response to environmental challanges.^32^, ^306^ As a result of this, tonality is a widespread feature of world music^11^ and is acquired implicitly.^330^, ^331^ Thus, it is probable that the sensitivity to pitch distribution is also a music‐specific kind of the experience–expectant plasticity, and tonality is therefore evolutionary in origin.^32^ Although older adults seem to refine their schematic expectations together with their lifelong musical experience, both younger and older adults are equally effective at the recognition of unexpected pitches,^332^ which also speaks for this explanation.

In contrast, the memory of melodic patterns seems to improve with the increasing experience of music and age,^333^ suggesting its reliance on experience‐dependent plasticity.^315^ This kind of developmental plasticity is lifelong among humans and allows us to learn completely new invented activities for which we did not evolve.^116^ This is not to say that sensitivity to melody is based solely on a general‐domain mechanism. Quite the opposite; the fact that people memorize sung melodies better than melodies played on instruments^334^ and that this advantage is independent of other biologically important signals coded in singers' timbre such as attractiveness,^335^ may suggest the evolutionary origin of vocalized pitch sequences as being part of the hominin communicative repertoire. However, the cultural variability of pitch sequences observed in contemporary musical cultures indicates the crucial role of vocal culture in the shaping of our sensitivity to melody (see Figure 4).

## CONCLUSION

As it has been presented, the suggested function of musical pitch as defensive signaling could have coexisted with other functions such as social bonding,^18^, ^19^, ^24^, ^32^, ^336^ signaling coalition,^20^, ^34^, ^44^ and free rider recognition.^33^ Rather than being mutually exclusive, they likely reinforced each other and created a synergistic loop that accelerated the evolution of musicality. One can argue, however, that hominins could have achieved the same functions without any of the abilities mentioned above. For instance, Merker raised the question of whether humans need music to bond at all, pointing out that nonhuman animals perform this task very well without music.^337^ The same logic can be used to question intraspecific defense signaling as an adaptive function of musical pitch. However, the hominin environment was characterized by increasing multilevel social complexity, which not only became a new specific source of selection, but also involved the creation of new niches with new challenges. Under such circumstances, adaptations that were effective in an environment of lower social complexity may not have been sufficient when hominin social complexity reached higher levels. Factors such as in‐group competition for resources, division of labor in a group, the level of cooperation, and limits of social interactions may have influenced the evolution of cognitive capabilities.^338^ For example, it has been indicated that the increase in social complexity could be one of the reasons for the selection of more complex communicative abilities.^76^, ^339^ Social complexity has also been claimed to be a source of the selection for abilities to sustain social cohesion.^340^ It is, therefore, reasonable to assume that in the case of a larger number of group members together with the changing aforesaid social factors, functions such as social bonding, signaling coalition or commitment, free rider recognition, as well as deterring foes could have also required new, more elaborate methods. The use of musical pitch as a defense signal is probably a type of an elaboration of more general signaling by means of rhythm synchronization.

The hypothesis that musical pitch may serve as a defensive function provides a new perspective on the evolution of musical behavior, integrating this function as a potential contributing factor without excluding other influences on human musicality. Importantly, this hypothesis is empirically testable through both observation and experimentation. As discussed, music continues to be employed in defensive contexts today and comparing pitch characteristics in such music with those in noncombat‐related music can clarify its specific defensive role. Additionally, human physiological and behavioral responses to variations in pitch can be measured, offering further insight into its adaptive significance. While further research is necessary to fully assess the evolutionary implications of musical pitch, this hypothesis expands the scope of inquiry and highlights previously unexplored pathways in the study of musicality.

## CONFLICT OF INTEREST STATEMENT

The author declares no conflicts of interest.

## PEER REVIEW

The peer review history for this article is available at https://publons.com/publon/10.1111/nyas.70000.
